# Causality and signalling of garden-path sentences

**DOI:** 10.1098/rsta.2023.0013

**Published:** 2024-03-18

**Authors:** Daphne Wang, Mehrnoosh Sadrzadeh

**Affiliations:** Department of Computer Science, University College London, London, UK

**Keywords:** sheaf theory, causality, contextuality, natural language ambiguities, psycholinguistics, garden path phenomena

## Abstract

Sheaves are mathematical objects that describe the globally compatible data associated with open sets of a topological space. Original examples of sheaves were continuous functions; later they also became powerful tools in algebraic geometry, as well as logic and set theory. More recently, sheaves have been applied to the theory of contextuality in quantum mechanics. Whenever the local data are not necessarily compatible, sheaves are replaced by the simpler setting of presheaves. In previous work, we used presheaves to model lexically ambiguous phrases in natural language and identified the order of their disambiguation. In the work presented here, we model syntactic ambiguities and study a phenomenon in human parsing called garden-pathing. It has been shown that the information-theoretic quantity known as ‘surprisal’ correlates with human reading times in natural language but fails to do so in garden-path sentences. We compute the degree of signalling in our presheaves using probabilities from the large language model BERT and evaluate predictions on two psycholinguistic datasets. Our degree of signalling outperforms surprisal in two ways: (i) it distinguishes between hard and easy garden-path sentences (with a p-value $<10^{-5}$), whereas existing work could not, (ii) its garden-path effect is larger in one of the datasets (32 ms versus 8.75 ms per word), leading to better prediction accuracies.

This article is part of the theme issue ‘Quantum contextuality, causality and freedom of choice’.

## 1. Introduction

Natural language ambiguities give rise to probability distributions and these can be studied in the mathematical framework of sheaf theory. Sheaf-theoretic models go all the way back to the work of Jean Leray during the second world war, and his formalization of fixed points of partial differential equations [1]. Later, it was Alexander Grothendieck that studied them in detail to unify different fields with mathematics [2]. In [3], Abramsky and Brandenburger showed that sheaf theory can also be applied to model the notion of contextuality in physics, and reason about paradoxes such as violations of Bell inequalities. In following works, Kishida *et al.* used sheaf theory to formalize a more general notion of paradox and in particular modelled famous logical paradoxes, such as the liar’s paradox [4,5]. Our previous work expanded on the latter and further showed that sheaf theory can model the manifold of interpretations arising from lexical ambiguities in natural language [6–8].

Natural language ambiguities are not limited to lexical ambiguities. They also occur in syntactic analysis, i.e. when a phrase can have multiple syntactic structures, or in discourse, where a pronoun or ellipsis marker can have more than one referent. In our preliminary work on the sheaf models of discourse, we showed that the ambiguities in the Winograd schema challenge, a pinnacle challenge of Natural Language Processing (NLP), can be extended to scenarios that exhibit quantum-like contextuality [9,10]. In the current paper, we show that sheaf theory can also model an interesting phenomenon in psycholinguistics, known as ‘the garden-path effect’. Garden-path sentences first appeared in [11] and sparked a research program on the biological and cognitive bases of human interaction patterns. The 1980s and 1990s saw a wave of psycholinguistic experiments aiming to classify these ambiguities and measure their cognitive dissonance levels. Recently, NLP researchers have found correlations between human reading times and statistics learnt by large language models [12]. However, these methods do not give as good results for garden-path sentences [13], and it remains an open problem to understand why that is the case.

Our choice of sheaf theory is motivated by its usage in quantum scenarios [3], which also motivated our previous study of lexical ambiguities [6–8]. The sheaf condition of quantum scenarios corresponds to the no-signalling condition in non-locality protocols. No-signalling can be imposed on physical systems, for instance by isolating the parties involved. This is much harder to impose on linguistic scenarios. In the absence of no-signalling, sheaf theory cannot straightforwardly be used. In order to remedy the situation, we previously used the setting of Contextuality-by-Default (CbD), which is able to analyse data coming from any scenario regardless of the no-signalling property [14,15]. We witnessed that linguistic data are able to show contextuality in the same ways quantum scenarios do (signalling property aside). More recently, the unsharpness of experimental apparatus inspired the sheaf theoretician to also factor in a *signalling fraction* in their models [9,10,16]. Working within this fraction, we were able to model discourse ambiguities and also find quantum contextual examples [9,10]. Another way of dealing with the presence of signalling is to study causal scenarios [17–19]. In previous work, we used causal models and the so-called *causal fraction* to further our study of lexical ambiguities [20]. In this paper, we focus on syntactic ambiguity and show that by building empirical models which imitate the incremental order of human sentence processing, the signalling fraction will correspond to the causal fraction associated with the reading order. We then make use of these fractions to predict human reading times of garden-path sentences and obtain improvement on existing research that uses an information theoretic measure known as surprisal.

## 2. Background

### (a) Garden-path sentences

In 1970, the psycholinguist Thomas Bever introduced a class of natural language sentences which, although unambiguous as a whole, are difficult for humans to parse due to local ambiguities [11]. A preliminary study of these sentences revealed that the difficulty arises from human biases in language usage, coming from a set of factors such as frequency, plausibility and primordiality: these biases lead the human subject down a ‘garden path’ when reading these sentences. Which one of these is the main factor remains debated amongst researchers; for instance, Bever’s view was that it is primordiality. The ambiguities studied by Bever were syntactic, in other words, they came from multiplicities of syntactic structures. Explicitly, a garden-path sentence is a sentence that has a single global syntactic structure but admits local syntactic ambiguities at some of its initial stages. In addition, a *garden-pathing* phenomenon is created in these sentences when the local structures which disambiguate the sentence have lower probabilities compared with the wrong ones. Examples of garden-path sentences are:

(1a) The employees understood the contract would change.
(1b) Because the employees negotiated the contract would change.

The locally ambiguous parts of these sentences are: ‘The employees understood the contract’ and ‘Because the employees negotiated the contract’. The (English-speaking) human biases about these initial structures tell them that—most probably—the verbs ‘understood’ and ‘negotiated’ have ‘the employees’ as subjects, and ‘the contract’ as objects. These come from two possibilities (i) either subject–verb–object is the most frequent syntactic form of English sentences, (ii) or the relation actor–action–object is a primordial humane semantic relation. As a result, the subject–verb–object construction has been observed/used much more than any other construction and the reader is eager to enforce it to the first three components of every sentence. As the reader reads on, they realize that, in fact, this hasty decision is wrong as the next part of the sentence, i.e. ‘would change’, does not fit in. In the case of (1a), a reanalysis reveals that the main verb takes the sentential (S) complement ‘the contract would change’ as an object instead of the noun phrase (NP) ‘the contract’. Such garden-path sentences, i.e. when an S is mistaken for an NP, are classified as NP/S. In the case of (1b), the reader eventually realizes that the main verb ‘negotiated’ has no (Zero) object, as opposed to the original analysis which designated the noun phrase (NP) ‘the contract’ as a complement. These sentences are classified as NP/Z. The above garden-path sentences are semantically equivalent to the following sentences, (2a) and (2b), which are much easier to understand. They will be referred to as the *unambiguous versions* of our garden-path sentences.

(2a) The employees understood *that* the contract would change.
(2b) Because the employees negotiated, the contract would change.

A major feature of garden-path sentences is a slowdown in reading time when entering the so-called *critical region* or *disambiguating region* of the sentence. In our examples, this is when reading the phrase ‘would change’. No slowdown is observed in the same region of the unambiguous versions. This difference in reading times is referred to as the *garden-path effect*. Different classes of garden path sentences, i.e. NP/S and NP/Z, result in different garden-path effects; for instance, NP/Z sentences are significantly harder to comprehend and therefore have a larger garden-path effect, than NP/S sentences [21,22].

### (b) Modelling garden-path with surprisal

Psycholinguistic studies have shown that one of the main factors influencing reading time is *predictability* of a word in context [23]. Words are read faster if found in a context that makes them predictable (e.g. ‘shark’ in the context ‘The coast guard had warned that someone had seen a’), than in contexts where they are not (e.g. ‘shark’ in the context ‘The zoo keeper explained that the lifespan of a’). In [12], it was shown that the relation between predictability and reading time is *logarithmic*. Formally speaking, *surprisal* is defined from the conditional probability of encountering a word w in the context $c=w_{1}\ldots w_{n}$ as:

$$SP(w|w_{1}\ldots w_{n})=-\log_{2}P[w\;|\;w_{1}\ldots w_{n}].$$

Also known as self-information, surprisal originates in Shannon’s theory of information [24], where it is defined as the *quantity of information* entailed by knowing the value X=w, where X is defined as a random variable selecting the next word in the context $w_{1}\ldots w_{n}$. The intuition is that a very predictable word is not surprising, and therefore does not carry out a lot of information; on the other hand, if a word is not predictable, it significantly increases the amount of information available to the reader. In psycholinguistics, surprisal is used as a predictor for reading time (RT), according to the following relation, pinned by [12]:

$$\mathrm{RT}(w|w_{1}\ldots w_{n})\propto SP(w|w_{1}\ldots w_{n}).$$

The above results were obtained by looking at eye-tracking times of a subset of the Dundee dataset, and self-paced reading times for subsets of the Brown corpus, both corpora containing naturalistic sentences. The idea of studying garden-path sentences using surprisal in fact predates these findings, and is attributed to the work of Hale [25]. In [25], he used the probabilities obtained from a probabilistic context-free parser to predict the existence of a garden-path effect. In the following work, empirical correlations between surprisal and self-paced reading times of garden-path sentences were also studied [13,26–28]. It was shown that, although the surprisal calculated using different language models is able to predict a garden-path effect, it consistently underestimates its magnitude. In addition, although predictions were mostly lower for NP/S than NP/Z [13,27], there was no statistical difference between the two; in fact, in [26], the average garden-path effect for NP/S sentences was lower than the one for NP/Z sentences.

### (c) Sheaf theory and quantum contextuality and causality

Sheaf theory has been used to model and reason about a fundamental phenomenon in quantum mechanics known as *contextuality*. Contextuality was first introduced by Kochen & Specker [29] as an extension of the principle of non-locality [30,31]. In [3], it was shown that in the language of sheaves, contextuality corresponds to the impossibility of finding a global section compatible with a family of local sections. In this framework, the possible local measurements are taken from a set X, and a compatibility relation is imposed on this set. This compatibility relation essentially encodes which measurements can be simultaneously made; we note that, interpreted as such, this relation is symmetric, i.e. a can be performed at the same time as b if b can be performed in the same time as a. For example, in the standard (2,2,2)-Bell scenario (i.e. consisting of two parties, each choosing between two measurements, and each measurement having two possible outcomes), we start from a set of measurements $X=\{a_{1},a_{2},b_{1},b_{2}\}$, where $I_{A}={\left\{a_{i}\right\}}_{i=1,2}$ corresponds to the choice of measurements available to Alice, and $I_{B}={\left\{b_{i}\right\}}_{i=1,2}$ is the set of measurements available to Bob. Then, any of Alice’s measurements in $I_{A}$ is compatible with any of Bob’s, i.e. $b\in I_{B}$. However, the measurements $a_{1}$ and $a_{2}$ are not compatible, as they cannot be performed simultaneously (and similarly for Bob’s measurements). Each of these measurements comes with a set of possible outcomes O.^1^ Then, given a set of compatible measurements U, we can see an event as associating outcomes with the measurements selected in U. This can be seen as a function:

s:U→O.

Formally speaking, these functions are modelled as the *presheaf of events*, i.e. a contravariant functor with the type $E:C^{op}\to Set$, where C is, in general, a subcategory of P(X) with the inclusion relation. The action of this *presheaf* on objects U gives us the set of all possible *assignments* or *functions* s:U→O. The action on morphisms f:U→V in C gives us all the *restrictions* of these assignments, namely:

2.1 resUV:E(V)→E(U) sV:V→O↦sV|U:U→O v↦ov u↦of(u)}

For example, in the (2,2,2)-Bell scenario, if Alice chooses to perform the measurement $a_{1}$ and obtains outcome x∈O, and Bob the measurement $b_{2}$ and obtains the outcome y∈O, then an event could be represented as the function:

$$s:U\to O::a_{1}\mapsto x;b_{2}\mapsto y.$$

In quantum mechanics, however, the outcomes of measurements are not generally deterministic, so instead of looking at events, it is more relevant to look at *the probability distributions* over all of the possible events. This is obtained by post-composing the event presheaf E with the distribution monad $D_{R_{+}}:Sets\to Sets$, which gives back a presheaf and is defined as follows:

$$\begin{array}{rl} \text{on objects:} & \;D_{R_{+}}:\text{Sets}\to \text{Sets}::U\mapsto \{d:U\mapsto U\to R_{+}\;|\;d\\ & \;\text{ probability distribution over }U\}\\ \text{on morphisms:} & \;\begin{array}{cccc} U\overset{f}{\to }V\mapsto & D_{R_{+}}(U) & \to & D_{R_{+}}(V)\\ & d_{U} & \mapsto & d_{V}\text{ s.t. }d_{V}(v)=\sum_{u\in f^{-1}(v)}d_{U}(u). \end{array} \end{array}$$

**Figure 1.**
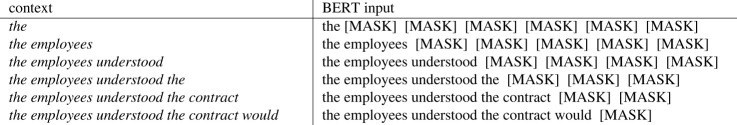
BERT inputs for the sentence. *The employees understood the contract would change*.

Given an object U∈C, an element of $D_{R}E(U)$ is called a *section* over U. This is where the notion of an *empirical model* comes in handy. These offer descriptions of the systems and each of them is the collection of probability distributions, over all of the possible events of a system. An example of an empirical model over the (2,2,2)-Bell scenario is depicted in figure 1. Formally speaking, given a set of objects M of C, an *empirical model* e is a collection of sections of the presheaf $D_{R}E$ which selects a single probability distribution for each context C∈M. The elements of M are the measurement contexts.

In the standard contextuality experiments, we are interested in studying the source of the correlations between contexts, i.e. choices of measurements, and their observed statistics. In order to isolate the source of potential correlations between the contexts and the outcomes, the standard practice is to limit the overall number of possible sources of such correlations. One type of correlation which can be eliminated in quantum experiments is communication, i.e. the *signalling* between Alice and Bob in the above example. In practice, this can be achieved by spatially isolating these parties. The consequence of such isolation, or lack of signalling, is that the marginal probability distributions do not depend on the choice of measurements of the other parties. In other words, for any set of inputs U, and any two sets of measurements $V,V^{\prime }$ compatible with all elements of U, we should have:

2.2 $$d_{U\cup V}{|}_{U}({\underline{o}}_{U})=d_{U\cup V^{\prime }}{|}_{U}({\underline{o}}_{U})$$

for all joint outcomes $\underline{o}$ over the measurements of U, where $d_{W}$ corresponds to the joint probability distribution corresponding with the choices of inputs W for any set W. The (2,2,2)-Bell scenario depicted in table 1 indeed satisfies this so-called *no-signalling* condition, since, for instance:

$$d_{\left\{a_{1},b_{1}\right\}}{|}_{\left\{a_{1}\right\}}(0)=d_{\left\{a_{1},b_{2}\right\}}{|}_{\left\{a_{1}\right\}}(0)=\frac{1}{2}.$$

Now, a system is said to be *non-contextual* if there exists a joint probability distribution over X which correctly restricts to all of the $d_{U}$’s. It can be shown that the example of figure 1 actually is *contextual*, i.e. such a global probability distribution cannot be defined. This is related to the formal definition of a sheaf. A *sheaf* is a presheaf $F:C^{op}\to Sets$ s.t. for every pair of objects U,V of C, the restriction morphisms from F(U) and F(V) to the intersection F(U∩V) agree, i.e. for $h_{U}\in F(U)$ and $h_{V}\in F(V)$, we have:

2.3 $$h_{U}{|}_{U\cap V}=h_{V}{|}_{U\cap V}.$$

We say that $h_{U}$ and $h_{V}$ are *compatible* (or consistent) whenever (2.3) is satisfied.

**Table 1.** An empirical model for the (2,2,2)-Bell scenario.

	(0,0)	(0,1)	(1,0)	(1,1)
$\left(a_{1},b_{1}\right)$	1/2	0	0	1/2
$\left(a_{1},b_{2}\right)$	3/8	1/8	1/8	3/8
$\left(a_{2},b_{1}\right)$	3/8	1/8	1/8	3/8
$\left(a_{2},b_{2}\right)$	1/8	3/8	3/8	1/8

In realistic experiments, the no-signalling condition does not usually hold; this can be due to unsharpness of the instruments [16] or simply finiteness of the measurements [15,16]. As a result, different frameworks have been developed to study contextuality in the presence of signalling. Examples of these are the Contextuality-by-Default framework [15] and the signalling fraction of the sheaf theoretic model [16], both of which create a measure of the signalling property of the system. A signalling system is then said to be contextual if the amount of signalling is not enough to make the system ‘classically explainable’. In sheaf-theoretic terminology, if every pair of sections in an empirical model satisfies the compatibility condition of (2.3), then the empirical model is said to be *no-signalling* or *consistent*. If the sections of an empirical model correspond to the statistics of a system, then the no-signalling condition is exactly the compatibility condition of (2.3). Given an empirical model e, which is not necessarily compatible, we define the *no-signalling fraction* NSF∈[0,1] as the maximal possible value of λ across all of the decompositions of e:

2.4 $$e=\lambda \cdot e_{\mathrm{NS}}+(1-\lambda )\cdot e^{\prime }$$

where $e_{\text{NS}}$ is a no-signalling empirical model (the multiplication here is understood as point-wise multiplication), and $e^{\prime }$ can be any empirical model. We then define the *signalling fraction* as:

2.5 SF=1−NSF.

This can be seen as the degree of incompatibility of an empirical model, as it measures the departure from a no-signalling, and hence locally compatible model.

In [17–19,32], this formulation of contextuality has been extended to scenarios where structured signalling is allowed, first by allowing sequential operations in [32], then by allowing *definitite causal orders* [17,19] and even *indefinite causal structures* [17,18]. In all of these studies, the introduction of causality was done by relaxing the symmetry property of the compatibility relation on X. In causal scenarios, a being compatible with b will mean that measuring a can (potentially) influence the outcome of the measurement made by b; we will write a⪯b. For example, starting from the same set of measurements as the (2,2,2)-Bell scenario, we can impose the conditions:

$$a_{1}⪯b_{1,2}\;\mathrm{and}\;a_{2}⪯b_{1,2}$$

in order to model the scenario where Alice can do her measurements before Bob. We can subsequently define the probability distributions as before, with the previous no-signalling condition being replaced by the *causality* condition; i.e. if U, V and $V^{\prime }$ are sets of measurements such that every element a∈U, b∈V and $b^{\prime }\in V^{\prime }$, we have a⪯b and $b⪯b^{\prime }$ (shorthanded to $U⪯V,V^{\prime }$), then:

$$d_{U\cup V}{|}_{U}({\underline{o}}_{U})=d_{U\cup V^{\prime }}{|}_{U}({\underline{o}}_{U})$$

for any joint outcome ${\underline{o}}_{U}$ over U. An example of such a causal scenario is shown in table 2. We can indeed see that for instance:

$$\begin{array}{rl} & d_{\left\{a_{1},b_{1}\right\}}{|}_{\left\{a_{1}\right\}}(0)=d_{\left\{a_{1},b_{2}\right\}}{|}_{\left\{a_{1}\right\}}(0)=\frac{6}{13}\\ \mathrm{and}\;\; & d_{\left\{a_{2},b_{1}\right\}}{|}_{\left\{a_{2}\right\}}(0)=d_{\left\{a_{2},b_{2}\right\}}{|}_{\left\{a_{2}\right\}}(0)=\frac{23}{65} \end{array}$$

but:

$$\begin{array}{rl} & 0=d_{\left\{a_{1},b_{1}\right\}}{|}_{\left\{b_{1}\right\}}(0)\neq d_{\left\{a_{2},b_{1}\right\}}{|}_{\left\{b_{1}\right\}}(0)=\frac{37}{65}\\ \mathrm{and}\;\; & \frac{59}{65}=d_{\left\{a_{1},b_{2}\right\}}{|}_{\left\{b_{2}\right\}}(0)\neq d_{\left\{a_{2},b_{2}\right\}}{|}_{\left\{b_{2}\right\}}(0)=\frac{191}{260}. \end{array}$$

Therefore, we have U⪯V but not the converse (i.e. V⪯̸U). Contextuality is, as before, defined as the impossibility of finding a global probability distribution over X which marginalizes to the original probability distributions. Similar to the signalling fraction, given a scenario endowed with an asymmetric compatibility condition, we define its *causal fraction* CausF as the minimal λ across all of the decompositions:

2.6 $$e=\lambda \cdot e_{\text{caus}}+(1-\lambda )\cdot e^{\prime }$$

where $e_{\text{caus}}$ is an empirical model consistent with the imposed compatibility relation.

**Table 2.** An empirical model for a causal scenario with $U=\{a_{1},a_{2}\}⪯V=\{b_{1},b_{2}\}$.

	(0,0)	(0,1)	(1,0)	(1,1)
$\left(a_{1},b_{1}\right)$	0	6/13	0	7/13
$\left(a_{1},b_{2}\right)$	24/65	6/65	7/13	0
$\left(a_{2},b_{1}\right)$	23/65	0	14/65	28/65
$\left(a_{2},b_{2}\right)$	23/260	69/260	42/65	0

The above notions are defined in the sheaf-theoretic framework of contextuality. In §3, we argue that they also provide a straightforward interpretation in terms of linguistic phenomena.

## 3 Methodology

We use sheaf theory to model the incremental process of reading and parsing, according to a popular theory known as parallel-ranking [22,33,34]. In this theory, as a human subject reads on, they keep track of and rank all possible local structures. These ranks are used to prune the tree of possibilities, by selecting the structures with the highest ‘ranks’. By analogy with sheaf theoretic models of quantum mechanics, we take the measurements to be words, their outcomes to be their grammatical structures and the measurement contexts to be linguistic contexts.

By analogy with the causal models, the compatibility relation is used to encode the linear order of the words in a sentence. The causality condition, if satisfied, then states that the process of building parses is purely incremental; as a new word is read, the reader will simply extend the existing graphs and assign probabilities to the new structures in a consistent way. In this section, we formalize these intuitions.

### (a) Sheaf-theoretic models of garden-path sentences

Given a garden-path sentence g, we denote its vocabulary by the set Σ(g) and refer to the monoid generated over it as $\Sigma {\left(g\right)}^{*}$; this monoid will be the base category C of our presheaf. Elements of $\Sigma {\left(g\right)}^{*}$ are seen as strings of words, i.e. phrases with elements in Σ(g), the monoid multiplication is concatenation, and its unit is the empty string. We endow this monoid with a partial order $-\leq -\subseteq \Sigma {\left(g\right)}^{*}\times \Sigma {\left(g\right)}^{*}$, and use it to denote the prefix order between the phrases, defined below:

$$m_{1}\leq m_{2}\;⟺\;m_{1}\;\text{is the initial subphrase of}\;m_{2}.$$

As an example, consider the garden-path sentence g = ‘The employees understood the contract would change’. Its vocabulary is the following set:

3.1 Σ(g)={employees, understood, the, contract, would, change}.

The monoid $\Sigma {\left(g\right)}^{*}$ contains all its subphrases and the following prefix orders between them:

$$\begin{array}{rl} \text{employees} & \;\leq \text{employees understood}\leq \text{employees understood contract}\\ \text{understood} & \;\leq \text{understood the}\leq \text{understood the contract.} \end{array}$$

To each $m\in \Sigma {\left(g\right)}^{*}$, we assign any *linguistic structure* that it might carry. This can be syntactic structure, semantic structure (e.g. its degree of plausibility), or pragmatic structure (e.g. the intentions or questions behind it). In this paper, our focus is on syntactic structure, and we choose to work with dependency relations [35]. These relations encode syntactic dependencies between words in a phrase or sentence. For instance, when a noun such as ‘employee’ is modified by an article, such as ‘the’, then the article depends on the noun. The modified word will then be called the *head* of the modifying word; this head is unique for any given word in a phrase. The set of all possible heads will be our set of outcomes. In a sentence of length n, each word can have n different possible heads. Hence, our set of outcomes is O={1,…,n} where n is the length of the sentence g.^2^ The maps s:m→O of the event presheaf assign syntactic structures to subphrases of a sentence. As an example, consider the sentence fragment ‘The employees understood’; a possible dependency parse for this fragment is as follows, where an arrow $w_{i}\to w_{k}$ indicates $w_{k}$ is the head of $w_{i}$:


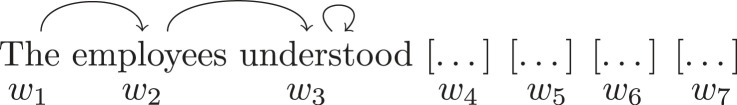

The function s: ‘The employees understood’ →O={1,…,7} assigns this structure to the sentence fragment as follows:

$$s=\{{\text{The}}_{1}\mapsto 2;{\text{employees}}_{2}\mapsto 3;{\text{understood}}_{3}\mapsto 3\}.$$

The restriction maps of our presheaf E are defined as in formula (2.1). For example, the restriction of the above grammatical structure from ‘The employees understood’ to ‘The employees’ is:


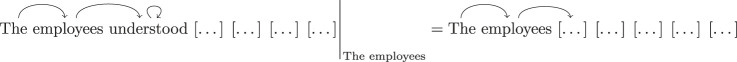

The function corresponding to it is $s{|}_{\text{The employees}}=\{{\text{The}}_{1}\mapsto 2;{\text{employees}}_{2}\mapsto 3\}$. The action of the distribution monad in turn sends sentence fragments to the set of *all* of the distributions over all of their grammatical structures. For example, for $m_{1}=$ ‘The employees’ and $m_{2}$ = ‘The employees understood’, we could have $d\in D_{R_{+}}E(m_{2})$ s.t.:


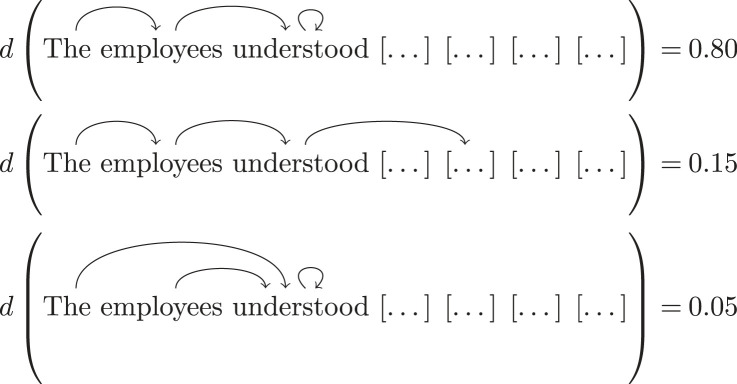

Their restriction morphisms are defined as follows. For $m_{1}=w_{1}\ldots w_{n}$ and $m_{2}=w_{1}\ldots w_{n}w_{n+1}\ldots w_{k}$ (i.e. $m_{1}\leq m_{2}$ in C), the restriction morphism from $D_{R_{+}}E(m_{2})\to D_{R_{+}}E(m_{1})$ takes any $d\in D_{R_{+}}E(m_{2})$ to the probability distribution s.t.:

3.2 $$d{|}_{m_{1}}(o_{1}\ldots o_{n})=\sum_{(o_{n+1}\ldots o_{k})\in O^{k-n}}d(o_{1}\ldots o_{n}o_{n+1}\ldots o_{k}).$$

In order to mimic the human reading process, we consider a *sequence* of empirical models, based on collections of subphrases ${\left\{M_{i}\right\}}_{1\leq i\leq n-1}$, where n is the length of the sentence. Elements of $M=\{m_{i},m_{i+1}\}$ are strings of lengths i and i+1, respectively, such that $m_{i}\leq m_{i+1}\leq g$. The sequences represent the evolution of the linguistic contexts. For instance, for g = ‘The employees understood the contract would change’, we have the following sequence:

$$\begin{array}{rl} M_{1} & \;=\{\text{The, The employees}\}\\ M_{2} & \;=\{\text{The employees, The employees understood}\}\\ M_{3} & \;=\{\text{The employees understood, The employees understood the}\}\\ & \;\vdots \\ M_{6} & \;=\{\text{The employees understood the contract would, }\\ & \;\text{The employees understood the contract would change}\} \end{array}$$

Recall that each of the empirical models consists of a pair of sections, i.e. a pair of probability distributions over grammatical structures; as we will see in §3c, these are obtained empirically. For example, a realization of $M_{3}=\{e_{\text{The employees understood}},e_{\text{The employees understood the}}\}$ in the above sequence is:
3.3

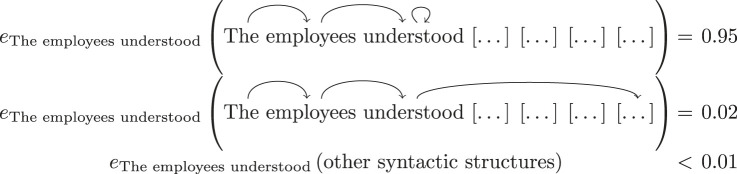

3.4

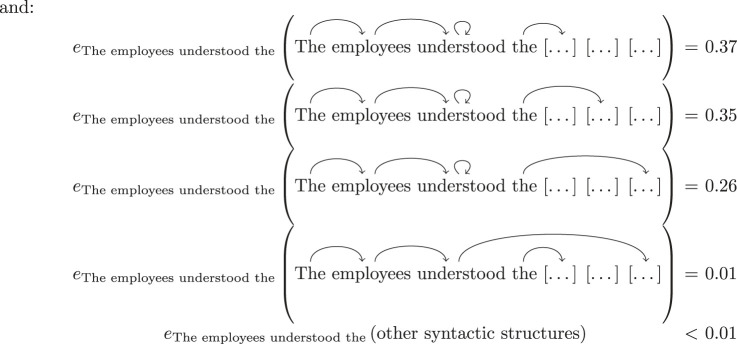

In this setting, the compatibility condition says that the maps that assign degrees of likelihood to syntactic structures of a common prefix of a subphrase, are compatible—in other words—agree with each other. This does not usually happen in data collected statistically. In the case of garden-path sentences, the amount of incompatibility is exacerbated, as the probabilities associated with certain syntactic structures dramatically change when entering the critical region. In other words, we should expect high signalling/non-causal fractions at the boundary of the critical region.

### (b) Analysis of the linguistic empirical models

In this section, we explore the properties of the linguistic models introduced above. We start by observing that the linguistic models can be seen as both a contextuality and a causality scenario. To see this, we first note that for each empirical model, one context includes exactly one less word than the other. As a result, we can w.l.o.g see empirical models as an {m,mw} scenario. For example, in the empirical model $M_{3}$ of §3a, we have m = ‘The employees understood’ and w = ‘the’. As a result, the compatibility relation of the linguistic models can be expressed as follows:

1. A symmetric relation analogous to measurements that can be simultaneously measured. In this case, we interpret m and w as being compatible, which means that m and w are somewhat parsed independently. In this interpretation, we have a situation similar to contextuality scenarios;
2. An asymmetric relation analogous to causal scenarios, where the compatibility relation read as m⪯w.

Both of these interpretations are possible, and very much related. However, the meanings of the quantity SF are subtly different. In the symmetric interpretation, SF quantifies how consistent the two probability distributions are. On the other hand, in the causal interpretation, the causal fraction is also a measure of the departure from a causal model imposed by the linear *reading* order; in other words, a high signalling fraction is evidence for the fact that the process of assigning a syntactic structure to a particular subphrase is not incremental, but instead should require information coming from the words situated *after* the phrase under consideration. For the rest of this paper, we will mostly focus on the signalling view of the models.

In addition, not all of the measurement scenarios are capable of hosting contextuality, and the structure of the M can be used to identify this. One can associate a simplicial complex with every M straightforwardly by defining for each C∈M with |C|=n an n+1-simplex, with a vertex for each local measurement, and identifying the faces of $C,C^{\prime }\in M$ corresponding to $C\cap C^{\prime }$. Vorob’ev’s theorem [36] then implies that if an empirical model is defined over a measurement context for which the topology of M does not contain a cycle, then it is non-contextual [37]. Since the linguistic contexts in each of our measurement scenarios are totally ordered, the geometric representations of the $M_{i}$’s do not contain any cycle and therefore cannot, by design, be contextual.

Finally, computing the signalling/causal fractions in a generic empirical model is not a trivial task, as it requires finding a solution to a linear optimization problem [16]. However, given the specific structure of our empirical models, it is possible to find an expression of the signalling fraction SF that can be calculated efficiently. This expression is shown in (A 10) and its proof can be found in appendix A.

**Proposition 3.1.**

*The signalling fraction can be computed via the following equation*:

3.5 $$SF=1-\sum_{o}\min(e_{mw}{|}_{m}(o),e_{m}(o)).$$

**Figure 2.**
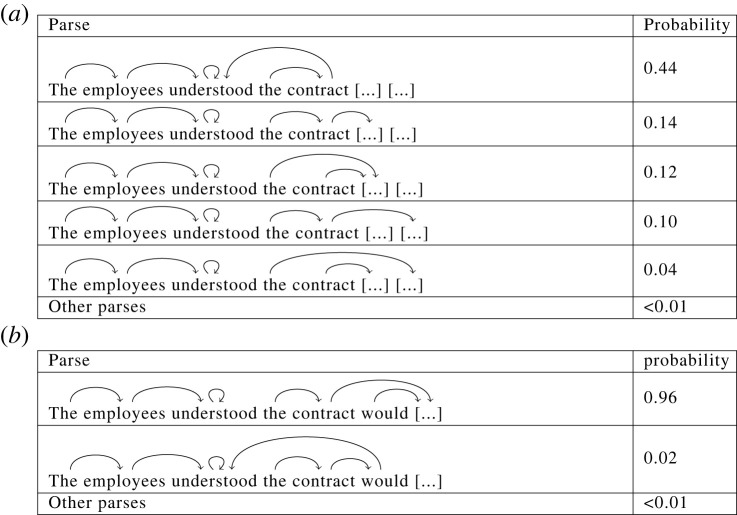
Example of an empirical model corresponding to $M_{5}$ in the sentence ‘The employees understood the contract would change’ (adapted from the empirical model obtained for ‘The faithful employees understood the technical contract would be changed’, which can be found in [41]). (*a*) Probability distribution for the context ‘The employees understood the contract’. (*b*) Probability distribution for the context ‘The employees understood the contract would’.

We argue that the signalling fraction can be seen as a measure of difficulty when assigning grammatical structure, in other words, parsing difficulty. This is motivated by the fact that SF can be seen as a measure of distance between probability distributions observed at different stages of the sentence. Therefore, the higher the syntactic fraction, the more a reader will have to readjust their mental representation of the grammatical structure. We can even say that since the contexts $m_{i},m_{i+1}\in M_{i}$ only differ by a single word, the signalling fraction of the empirical model $e_{i}$ becomes related to the difficulty of understanding the extra word. For example, for the empirical model $M_{3}$ defined in (3.3) and (3.4), we obtain a signalling fraction of ${SF}_{3}=0.05$, hence showing that the word ‘the’ at the end of the fragment ‘The employees understood the’ is not difficult to parse. On the other hand, if we calculate the signalling fraction for the empirical model $M_{5}$ (see figure 2 in appendix B), for the signalling fraction we have ${SF}_{5}=0.79$, which reflects the fact the parsing the word ‘would’ is quite difficult.

### (c) Data collection using deep neural network language models

In order to obtain probabilities of different syntactic structures of subphrases of a garden-path sentence, we need to input these subphrases to an automatic dependency parser and we use the state-of-the-art dependency parser spaCy [38]. As spaCy only assigns syntactic structures to completed sentences, we need to first come up with completions of each subphrase under consideration. To this end, we use the *masking* tool of another state-of-the-art NLP tool: the Bidirectional Encoder Representations from Transformers language model, also known as BERT [39,40]. BERT is trained on a word-in-context prediction task, that is, given a sequence of words where one or more words are *masked*, the neural network is trained to predict the values of those masks and provide a degree of likelihood for each of its predictions. We use BERT to obtain completions of phrases and their probabilities according to the following steps:

1. Given a subphrase of a garden-path sentence, we turn it into a complete sentence by masking all of the remaining words of the g, see figure 1 for an example.
2. BERT provides a list of predictions of the completion of the subphrases^3^ and a *logit* score s for each of these predictions, which is meant to rate the likelihood of each prediction. The common practice in NLP is to use the logistic function $p={\text{e}}^{s}/\left(1+{\text{e}}^{s}\right)$ to turn these scores into probabilities.
3. We then use spaCy to parse each of the predictions provided by BERT. In order to obtain the grammatical structure of the specific subphrase we are working with, we restrict the full parse to the words included in that subphrase using the restriction maps from the presheaf E (see equation (2.1)).
4. The probability of each such (partial) parse is obtained by summing up all the BERT-prediction probabilities which restrict to the same parse. For example, consider the predictions for the continuations of ‘The employees understood’:


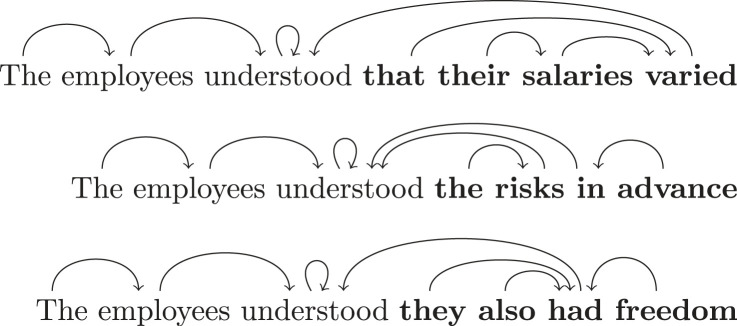

These lead to the same partial parse when restricted to the context ‘The employees understood’, namely:


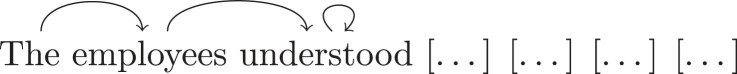

The resulting probability distributions are subsequently used in empirical models to calculate signalling fractions.

We work with different variations of BERT and spaCy. For BERT, first and foremost we worked with its original version bert-base-cased, which has 110 million parameters and was trained on Toronto Book Corpus and the English Wikipedia, both of which are cased, i.e. distinguish between lower and upper case letters. Uncased versions of the same algorithm exist and were developed for purposes of cross-lingual learning. Secondly, we worked with bert-large-cased, which has 340 million parameters. Finally, we worked with distilBERT, which only has 40% of the parameters of the original BERT model, but runs 60% faster while preserving 95% of its performance accuracies in language understanding tasks. For spaCy, we worked with its small and large versions, called en_core_web_sm and en_core_web_lg. Both models are trained using convolutional neural networks on web text consisting of blogs, news and comments. The difference between them is that the small version does not have any word vectors whereas the large one has a word vector table with 500k unique 300-dimensional vectors. We also worked with a newer version of spaCy,en_core_web_trf, which has no word vectors but is trained using state-of-the-art transformer-based neural networks. We compare the values of SF and the accuracies of their predictions between the different versions of these tools. The consensus in the machine learning community is that models trained on a larger corpus with a larger set of parameters, including the dimensionality of vectors, should be more accurate. We work with their different variants to obtain a better understanding of the robustness of our results.

## 4. Results

We work with the datasets of Sturt *et al.* [21] and the dataset of Grodner *et al.* [22]. Each garden-path sentence of each of these datasets is paired with its disambiguated version. The Sturt *et al.* dataset consists of 32 ambiguous NP/S, and 32 ambiguous NP/Z sentences. Taking the disambiguated versions of each sentence into account, this gives a total of 128 sentences. Sturt *et al.* divide each of their sentences, whether garden-path or unambiguous, into four regions, see table 3*a*, where the penultimate region is the *critical region*. They report reading times for each of the regions; these reading times are then averaged over the different sentences of the same type (i.e. NP/S ambiguous, NP/S unambiguous, NP/Z ambiguous or NP/Z unambiguous). The dataset of Grodner *et al.* consists of 40 ambiguous NP/S and 40 ambiguous NP/Z sentences and their disambiguated variants, i.e. a total of 160 sentences. Similar to the Sturt *et al.* dataset, each sentence is divided into regions, but in this case, the regions are different for the different types of sentences, see table 3*b*. Grodner *et al.* reports an average word-by-word reading times for each region, averaged again across the different sentences of the same type.^4^ The reading times of these dataset are in table 10 appendix C(a).

**Table 3.** Example of different regions of different datasets. The critical regions are in italics.

	regions					
(*a*) Sturt *et al.* dataset						
NP/S (ambiguous)	the faithful employees	understood the technical contract	*would be changed*	very soon		
NP/S (unambiguous)	the faithful employees	understood that the technical contract	*would be changed*	very soon		
NP/Z (ambiguous)	because the employees	negotiated the technical contract	*would be changed*	very soon		
NP/Z (unambiguous)	because the employees	negotiated, the technical contract	*would be changed*	very soon		
(*b*) Grodner *et al.* dataset						
NP/S (unmod., ambiguous)	the employees	understood	the contract	*would be changed*		
NP/S (unmod., unambiguous)	The employees	understood	that	the contract	*would be changed*	
NP/S (mod., ambiguous)	the employees	who initiated the strike	understood	the contract	*would be changed*	
NP/S (mod., unambiguous)	the employees	who initiated the strike	understood	that	the contract	*would be changed*
NP/Z (unmod., ambiguous)	even though the band	left	the party	*went on for* […]		
NP/Z (unmod., unambiguous)	Even though the band	left,	the party	*went on for* […]		
NP/Z (mod., ambiguous)	even though the band	which played funk music	left	the party	*went on for* […]	
NP/Z (mod., unambiguous)	even though the band	which played funk music	left,	the party	*went on for* […]	

The data collection procedures differed in [21,22] and one can argue that the reading times they studied are fundamentally different. We did not use standard methods to unify these datasets to avoid the systematic error caused by the human/computer reaction times. These are induced by the change of stimuli (i.e. when a new word or phrase is displayed on screen). Assuming that this time is constant for a given participant, it occurs once per region in the region-by-region setting of Sturt *et al*., but multiple times per region in the word-by-word setting of Grodner *et al.* With regard to this, what we are investigating is the assumption that only the reading difficulty of a given word is affected by SF, all other factors are kept constant. Then, in the case of the word-by-word setting, we are mimicking the averaging procedure of Grodner *et al*. [22] and study the correlation between the average of the word-by-word SF, across a given region, and all the sentences of this type. On the other hand, in the case of the region-by-region setting, we are investigating the correlation between the *sum* of the word-by-word SF in a given region and the corresponding region’s reading time.

The empirical models obtained for all of the variants of the BERT and spaCy tools for all of the sentences of these datasets are available online at [41].

### (a) Correlations between SF and reading times

We test the degree of correlation between SF and reading times and find a strong positive correlation. The resulting Pearson’s ρ coefficients and associated p-values are in table 4. For both datasets and regardless of the choice of BERT and spaCy, Pearson’s coefficients are statistically significantly above 0. They are also fairly high; in particular, for the dataset of Sturt *et al.* [21]. By considering all of the data obtained from all of the BERT and spaCy combinations, we obtain the following linear regression equations:

4.1 $${\mathrm{RT}}_{\text{Sturt}}(\text{region})=295\sum_{w\in \text{region}}SF(w)+664\;\mathrm{ms}$$

and

4.2 $${\mathrm{RT}}_{\text{Grodner}}(w)=77\;SF(w)+381\;\mathrm{ms}.$$

The individual linear regression equations for each of the specific BERT/spaCy models combinations were also solved and are provided in appendix C(b) table 11. All coefficients (the individual ones, as well as the ones of (4.1) and (4.2)) were within each other’s standard error. We used the individual linear regression models of table 11 for our prediction task, presented in the next paragraph.

**Table 4.** Pearson’s ρ coefficients and associated p-values (in brackets) between SF and reading times. The statistically significant p-values are in italics. Only the p-values marked with ${\;}^{†}$ are not statistically significant after Bonferroni correction.

		BERT model		
		distilbert	bert-base	bert-large
(*a*) Sturt *et al.* dataset				
spaCy model	en_core_web_sm	0.64 (*0.008*)	0.80 (*0.0002*)	0.79 (*0.0003*)
	en_core_web_lg	0.63 (*0.009*)	0.79 (*0.0003*)	0.78 (*0.0003*)
	en_core_web_trf	0.67 (*0.004*)	0.78 (*0.0004*)	0.76 (*0.0006*)
(*b*) Grodner *et al.* dataset				
spaCy model	en_core_web_sm	0.55 (*0.0004*)	0.35 ($0.03^{†}$)	0.56 (*0.0002*)
	en_core_web_lg	0.52 (*0.0008*)	0.37 ($0.02^{†}$)	0.48 (*0.002*)
	en_core_web_trf	0.53 (*0.0006*)	0.36 ($0.02^{†}$)	0.44 (*0.006*)

#### (i) Detecting a garden-path effect

Given that SF is correlated with reading times, we expect to observe a difference between SF of the garden-path sentences versus SF of their unambiguous variants, in their critical regions. This is indeed the case, see table 5. As we can see, the average difference in SF is positive. In order to test whether the departures from 0 are significant, in table 6, we do a t-test for the null hypothesis that the differences between SF in garden-path sentences and SF in the corresponding unambiguous variants are on average 0. As we can see, the p-values are quite low in most cases, and they approach 0 for the BERT and spaCy models with a larger set of parameters.^5^

**Table 5.** Average difference of SF between garden-path sentences and their unambiguous variants with standard deviation. Note: for the dataset of Sturt *et al*. [21], the sum of SF over the critical regions are quoted, while for the dataset of Grodner *et al*. [22], the mean of SF is shown.

		BERT model		
		distilbert	bert-base	bert-large
(*a*) Sturt *et al.* dataset				
spaCy model	en_core_web_sm	00.07±0.18	0.15±0.16	0.16±0.17
	en_core_web_lg	00.03±0.14	0.15±0.18	0.17±0.17
	en_core_web_trf	00.18±0.20	0.29±0.18	0.29±0.19
(*b*) Grodner *et al.* dataset				
spaCy model	en_core_web_sm	00.02±0.11	0.03±0.10	0.05±0.11
	en_core_web_lg	0.007±0.08	0.05±0.08	0.06±0.10
	en_core_web_trf	00.06±0.06	0.07±0.06	0.06±0.07

**Table 6.** p-values associated with the t-tests evaluating whether the unambiguous and ambiguous SF are the same. The lower these numbers, the more significant the results. The statistically significant results are highlighted in italics.

		BERT model		
		distilbert	bert-base	bert-large
(*a*) Sturt *et al.* dataset				
spaCy model	en_core_web_sm	*0.005*	$<10^{-8}$	$<10^{-9}$
	en_core_web_lg	0.07	$<10^{-7}$	$<10^{-10}$
	en_core_web_trf	$<10^{-9}$	$<10^{-18}$	$<10^{-17}$
(*b*) Grodner *et al.* dataset				
spaCy model	en_core_web_sm	0.13	*0.01*	*0.0004*
	en_core_web_lg	0.46	$<10^{-5}$	$<10^{-5}$
	en_core_web_trf	$<10^{-10}$	$<10^{-13}$	$<10^{-9}$

#### (ii) Garden-path effect predictions

**Figure 3.**
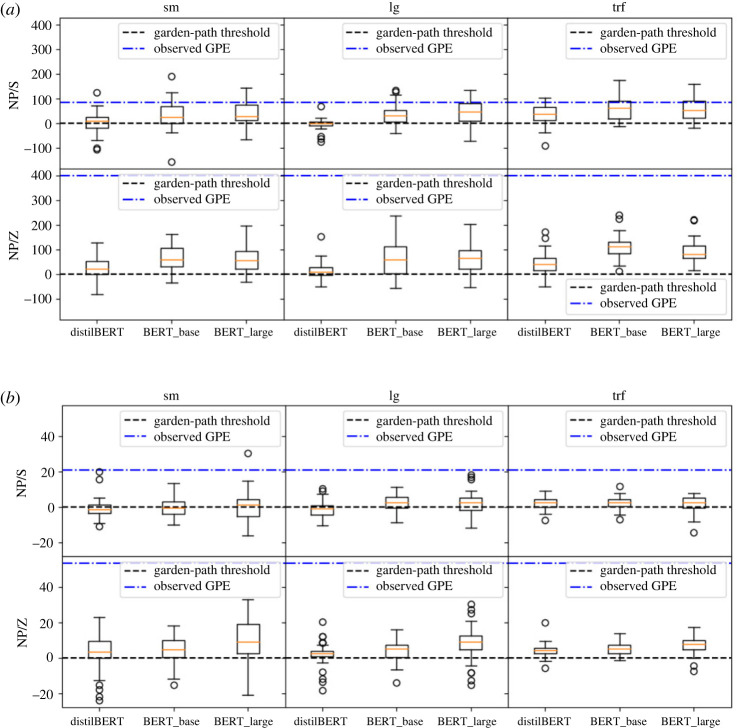
Garden-path effect predictions. The spaCy models are labelled at the top and the BERT models at the bottom. (*a*) Sturt *et al.* dataset. (*b*) Grodner *et al.* dataset.

We used each of the individual regression equations of table 11 to predict the reading times resulting from SF. We predict the garden-path effect by taking the difference between predictions for garden-path sentences and their unambiguous variants over the critical region. For all BERT and spaCy models, our SF predictions underestimate the garden-path effects, in particular for NP/Z sentences. However, the predicted effects increase as the BERT and spaCy models get larger. The average predictions are depicted in table 7 (see also figure 3 in appendix C(c) for more details). On the whole, bert-base and bert-large provided better predictions than distilbert; similarly the en_core_web_trf outperformed other variants of spaCy. Some of the combinations using smaller models did not provide accurate predictions and in fact predicted the wrong trend (i.e. predicted that garden-path sentences were read *faster* than their unambiguous analogues).

**Table 7.** Average predicted garden-path effects for NP/S and NP/Z sentences in milliseconds (the type of sentences is shorthanded in brackets: S for NP/S and Z for NP/Z).

		BERT model		
		distilbert	bert-base	bert-large
(*a*) Sturt *et al*. The human garden-path effect is 87 ms for NP/S sentences and 400 ms for NP/Z sentences				
spaCy model	en_core_web_sm	4.41(S);30.0(Z)	33.0(S);69.6(Z)	41.6(S); 65.4(Z)
	en_core_web_lg	−1.89(S);17.5(Z)	33.5(S);65.8(Z)	47.1(S); 63.4(Z)
	en_core_web_trf	35.2(S);44.9(Z)	60.9(S);110(Z)	62.6(S); 93.6(Z)
(*b*) Grodner *et al*. The human garden-path effect is 21 ms for NP/S sentences and 53.5 ms for NP/Z sentences				
spaCy model	en_core_web_sm	−0.51(S);3.90(Z)	−0.30(S);4.41(Z)	0.73(S); 9.36(Z)
	en_core_web_lg	−1.00(S);2.07(Z)	2.25(S);4.39(Z)	2.15(S); 8.52(Z)
	en_core_web_trf	2.73(S);4.71(Z)	2.62(S);5.57(Z)	1.90(S); 7.41(Z)

#### (iii) Comparison of NP/S and NP/Z predictions

Now that we have predicted the garden-path effect for NP/S and NP/Z sentences, we are interested to see whether SF distinguishes between them. We performed t-tests to verify this hypothesis. The p-values are in table 8. For most of the BERT and spaCy variants, there is a statistically significant difference in reading time predictions^6^ (apart from a couple of exceptions in both datasets). This shows that SF is able to witness the increase of difficulty induced by having an NP/Z ambiguity type. As before, bert-base or bert-largse and the en_core_web_trf variants distinguished the NP/S and NP/Z sentences more accurately.

**Table 8.** p-values associated with the t-test evaluating whether the garden-path effects obtained from SF for NP/S and NP/Z are sampled from the same distribution. The statistically significant p-values are highlighted in italics. Only the p-values marked with ${\;}^{†}$ are not statistically significant after Bonferroni correction.

		BERT model		
		distilbert	bert-base	bert-large
(*a*) Sturt *et al.* dataset				
spaCy model	en_core_web_sm	${\text{0.03}}^{†}$	*0.01*	0.09
	en_core_web_lg	${\text{0.02}}^{†}$	${\text{0.04}}^{†}$	0.24
	en_core_web_trf	0.39	*0.0001*	*0.01*
(*b*) Grodner *et al*. dataset				
spaCy model	en_core_web_sm	${\text{0.04}}^{†}$	*0.002*	*0.001*
	en_core_web_lg	${\text{0.02}}^{†}$	0.09	*0.001*
	en_core_web_trf	${\text{0.03}}^{†}$	*0.0006*	$<{\text{10}}^{-\text{5}}$

### (b) Comparison with surprisal

Schijndel & Linzen [13] generated a linear regression model of reading times as a function of surprisal, and used it to predict garden-path effects. Similar to us, they observed that surprisal underestimates the garden-path effects, but different from us, they could not distinguish between the NP/S and NP/Z sentences [13,26,27].

Although statistical tests are not quoted in [13], our model clearly outperforms the results obtained from surprisal and our predictions for NP/Z sentences are significantly higher than for NP/S sentences, see table 9. The SF values are closer to the observed human garden-path effects in the Sturt *et al.* dataset [21], and in the Grodner *et al.* dataset [22], they are comparable to the predictions provided by surprisal. For instance, the SF values of the NP/S sentences of [21] predict a slowdown of about 63 ms, whereas surprisal only predicts a slowdown of 24 ms at best (the human effect is 87 ms).

**Table 9.** Optimal (averaged) garden-path effect predictions. The best predictions are denoted in italics.

		prediction (ms)		
		SF	SP [13]	observed (ms)
Sturt *et al.*	NP/S	*62.6*	24	87
	NP/Z	*110*	30	400
Grodner *et al.*	NP/S	2.73	*7*	21
	NP/Z	8.52	*10*	53.5

Even though our usage of SF stemmed from similar motivations to those for surprisal, it is not clear whether they are mathematically related. The reason for the better performance of SF is that surprisal, as used in [13], mostly focuses on lexical items, whereas for the SF quantity described here, syntactic structures are first-class citizens. Only very recently (this year), the role of syntactic structure in conjunction with surprisal has come to light: in [28], it was shown that syntactic surprisal performs slightly better than pure lexical surprisal, but still falls short when distinguishing NP/S from NP/Z and the differences in garden-path effects. The results of [28] and the ones presented here motivate the hypothesis that syntactic structures are the main deciding factor in the difficulty of garden-path sentences. Another aspect of our work, which may have led to more accurate results, is that our model is able to take long-distance dependencies into account, whereas surprisal is not.

## 5. Conclusion and discussion

In this work, we constructed a sheaf-theoretic model of human sentence processing, inspired by similar models in quantum contextuality and causality. The base category of our sheaf was a partial order category of sentence fragments, where the signalling and causal fractions coincided. We applied our model to formalize and reason about a challenge known as the garden-path effect, which happens in certain sentences with local syntactic ambiguity. Garden-path sentences have higher reading times in comparison to their locally unambiguous versions. We showed that the signalling/causal fraction correlated well with human reading times. Using this correlation, we predicted reading times with a par (in the Grodner *et al.* [22] dataset) and better (in the Sturt *et al.* [21] dataset) accuracies than recent research in NLP that uses an information-theoretic measure called ‘surprisal’. Furthermore, we could observe significantly different predictions for easy and hard garden-path sentences; to date, surprisal-based models have not been able to do so. The better performance of the sheaf-theoretic model is that it takes both linguistic structure and lexical statistics into account, whereas surprisal-based models are only able to focus on one or the other.

Factors other than syntactic structure and lexical statistics are argued for when analysing garden-path sentences, e.g. semantic plausibility [42,43] and pragmatic concerns [21]. In this work, we only considered syntax. However, sheaf theory offers tools for modelling semantic plausibility, e.g. via composition with a Boolean truth-value functor. At this time, it is not clear to us how pragmatics concerns can be modelled. Furthermore, we only modelled forward-looking human processes. Psycholinguistic research shows that humans require backtracking in order to process garden-path sentences. We believe backtracking can be formalized in our sheaf-theoretic model, e.g. by using different compatibility relations. A third limitation of our work has been the differences between the datasets available to us. Indeed, the reading times used for doing the linear regression were only averages of reading times across different sentences and different participants; and this caused discrepancies in our results. We plan to use more detailed datasets such as a recent one released in [27] in order to confirm our results.

## Data Availability

This article has no additional data.

## Footnotes

1 Wlog, we can assume that O is the same for any choice of measurement.

2 If we were to consider sentences of any length, we could also take O=N.

3 These predictions are not always words and may include punctuations. To avoid complications, we only include the text predictions by dropping the punctuation and renormalizing the probability distribution.

4 Half of the NP/S and NP/Z sentences (20 sentences each) were labelled as ‘modified’, as they contained a descriptive noun-phrase attached to the subject of the main verb, whereas the rest of the sentences were labelled as ‘unmodified’. This distinction was made since the authors were investigating whether adding modifying noun-phrase would alter the misanalysis difficulty. Since we are not interested in this distinction in the current work, we will treat both (un)modified versions as the same.

5 One may also note that, although the results appear to be better as the BERT and spaCy models get better, the highest correlation and, respectively, the lowest p-value are not necessarily obtained for the combination using both of the the BERT and spaCy variants with the largest number of parameters. This could be due to statistical variations. This remark will also apply to the subsequent results as well.

6 In the case of the Sturt *et al.* dataset, most of the p-values are no longer significant after Bonferroni correction with significance level α=0.05; however, the p-values do remain low and only one entry of the table will no longer be statistically significant after Bonferroni correction at significance level α=0.1, which may be more relevant in this study anyway given the noisiness of the data.

## Appendix A. Proof of proposition 3.1

Each of our contexts includes exactly one less word than the next one. As a result, given a pair of successive contexts, we can w.l.o.g consider the 2-context scenario as an {m,mw} scenario. The no-signalling condition of the model is then as follows:

A 1 $$e_{mw}{|}_{m}=e_{m}.$$

Given an arbitrary 2-context empirical model as above, we want to find the following decomposition:

A 2 $$e=NSF\cdot e_{\mathrm{NS}}+SF\cdot e^{\prime }$$

where $e_{\text{NS}}$ is the maximum possible across all such decompositions. Let us assume, as above, that m is a single ‘observable’ with possible outcomes in $O^{m}=\{0,\ldots ,n^{|m|}-1\}$, where |m| is the number of words in the context m. Our first goal is to find a distribution for m in $e_{\text{NS}}$, which satisfies the following for all $o_{i}\in O^{m}$:

A 3 $$NSFe_{\mathrm{NS},m}(o_{i})\leq \min(e_{mw}{|}_{m}(o_{i}),e_{m}(o_{i})).$$

From the above, it follows that

A 4 $$\sum_{o_{i}}NSFe_{\mathrm{NS},m}(o_{i})=NSF\leq \sum_{o_{i}}\min(e_{mw}{|}_{m}(o_{i}),e_{m}(o_{i})).$$

One can always construct an empirical model $e_{\text{NS}}$ s.t.:

A 5 $$\sum_{o_{i}}\min(e_{mw}{|}_{m}(o_{i}),e_{m}(o_{i}))e_{\mathrm{NS}}\leq e.$$

In order to see this, first observe that the probability distribution of $e_{\text{NS},m}$ can be constructed by first relabeling the outcomes to $o_{i_{k}}$ for $0\leq k\leq n^{|m|}-1$ s.t. for $N=n^{|m|}-1$, the following holds:

A 6 $$\begin{array}{rl} \min(e_{mw}{|}_{m}(o_{i_{0}}),e_{m}(o_{i_{0}})) & \;\leq \min(e_{mw}{|}_{m}(o_{i_{1}}),e_{m}(o_{i_{1}}))\\ & \;\leq \cdots \leq \min(e_{mw}{|}_{m}(o_{i_{N}}),e_{m}(o_{i_{N}})). \end{array}$$

Then, we take σ to be $\sum_{o_{i}}\min(e_{mw}{|}_{m}(o_{i}),e_{m}(o_{i}))$ and set:

A 7 $$e_{\mathrm{NS},m}(o_{i_{0}})=\frac{\min(e_{mw}{|}_{m}(o_{i_{0}}),e_{m}(o_{i_{0}}))}{\sigma }.$$

We can then inductively define:

A 8 $$e_{\mathrm{NS},m}(o_{i_{k}})=\min\left(\frac{\min(e_{mw}{|}_{m}(o_{i_{0}}),e_{m}(o_{i_{0}}))}{\sigma },1-\sum_{j=0}^{k-1}e_{\mathrm{NS},m}(o_{i_{j}})\right).$$

From this definition, we know that for all k, we have the following:

A 9 $$\sigma e_{\mathrm{NS},m}(o_{i_{k}})\leq e_{mw}{|}_{m}(o_{i_{k}}),e_{m}(o_{i_{k}}).$$

In addition, the above forms a valid probability distribution as, if there exists a k s.t. $e_{\text{NS},m}(o_{i_{k}})=1-\sum_{j=0}^{k-1}e_{\text{NS},m}(o_{i_{j}})$, then $e_{\text{NS},m}(o_{i_{k}^{\prime }})=0$ for all $k^{\prime }>k$ and therefore:

A 10 $$\sum_{k}e_{\mathrm{NS},m}(o_{i_{k}})=1.$$

Similarly, if for all k, $e_{\text{NS},m}(o_{i_{k}})=\min(e_{mw}{|}_{m}(o_{i_{0}}),e_{m}(o_{i_{0}}))/\sigma$, then by definition of σ, we also have (A 10). We extend this probability distribution to an empirical model over {m,mw}, by defining:

A 11 $$e_{\mathrm{NS},mw}(o_{m},o_{w})=e_{mw}(o_{m},o_{w})\frac{e_{\mathrm{NS},m}(o_{m})}{e_{mw}{|}_{m}(o_{m})}.$$

It is now easy to show that $e_{\text{NS},mw}{|}_{m}=e_{\text{NS},m}$, and in addition, we have:

A 12 $$\sigma e_{\mathrm{NS},mw}(o_{m},o_{w})=e_{mw}(o_{m},o_{w})\frac{\sigma e_{\mathrm{NS},m}(o_{m})}{e_{mw}{|}_{m}(o_{m})}\leq e_{mw}(o_{m},o_{w}).$$

As a result of the above calculations, the signalling fraction can be computed as follows:

A 13 $$SF=1-\sum_{o}\min(e_{mw}{|}_{m}(o),e_{m}(o)).$$

## Appendix C. Details of the garden-path effects

**(a) Reading times collected in [21,22]**

**Table 10.** Reading times (in milliseconds) reported in [21,22].

	regions					
(*a*) Sturt *et al*. dataset						
NP/S (ambiguous)	990	1183	877	771		
NP/S (unambiguous)	981	1282	790	768		
NP/Z (ambiguous)	914	1269	1335	848		
NP/Z (unambiguous)	998	1384	935	832		
(*b*) Grodner *et al.* dataset						
NP/S (unmod., ambiguous)	397	467	412	424		
NP/S (unmod., unambiguous)	393	460	431	396	410	
NP/S (mod., ambiguous)	392	415	449	401	419	
NP/S (mod., unambiguous)	398	413	471	393	388	391
NP/Z (unmod., ambiguous)	452	402	382	452		
NP/Z (unmod., unambiguous)	400	452	402	383		
NP/Z (mod., ambiguous)	433	407	464	415	432	
NP/Z (mod., unambiguous)	405	400	494	448	395	

**(b) Individual linear regression models**

**Table 11.** Individual linear regression models (in milliseconds).

	distilbert	bert-base	bert-large
(*a*) Sturt *et al.* dataset			
en_core_web_sm	263(±85)∑SF+690(±111)	352(±72)∑SF+615(±87)	333(±69)∑SF+612(±89)
en_core_web_lg	250(±82)∑SF+710(±108)	340(±71)∑SF+627(±88)	321(±68)∑SF+621(±89)
en_core_web_trf	223(±66)∑SF+734(±91)	297(±64)∑SF+682(±79)	273(±62)∑SF+684(±82)
(*b*) Grodner *et al.* dataset			
en_core_web_sm	87(±22)SF+376(±12)	68(±31)SF+386(±16)	108(±27)SF+367(±13)
en_core_web_lg	74(±20)SF+382(±11)	70(±29)SF+386(±15)	90(±27)SF+376(±14)
en_core_web_trf	65(±17)SF+385(±10)	60(±26)SF+390(±13)	75(±26)SF+383(±13)

**(c) Predicted garden-path effects for different models**
